# Evaluating the success of functional restoration after reintroduction of a lost avian pollinator

**DOI:** 10.1111/cobi.13892

**Published:** 2022-04-07

**Authors:** Caitlin E. Andrews, Sandra H. Anderson, Karin van der Walt, Rose Thorogood, John G. Ewen

**Affiliations:** ^1^ Department of Zoology University of Cambridge Cambridge UK; ^2^ Institute of Zoology Zoological Society of London London UK; ^3^ School of Biological Sciences University of Auckland Auckland New Zealand; ^4^ Ōtari Native Botanic Garden and Wilton's Bush Reserve Wellington New Zealand; ^5^ Helsinki Institute of Life Science (HiLIFE) University of Helsinki Helsinki Finland; ^6^ Research Program in Organismal and Evolutionary Biology, Faculty of Biological and Environmental Sciences University of Helsinki Helsinki Finland

**Keywords:** mutualismos, polinización realizada por animales, polinizador, recuperación del ecosistema, restauración ecológica, retorno a la vida silvestre, reubicación para la conservación, animal‐mediated pollination, conservation translocation, ecological restoration, ecosystem recovery, mutualisms, plant–pollinator interactions, rewilding

## Abstract

Conservation translocation is a common method for species recovery, for which one increasingly frequent objective is restoring lost ecological functions to promote ecosystem recovery. However, few conservation translocation programs explicitly state or monitor function as an objective, limiting the ability to test assumptions, learn from past efforts, and improve management. We evaluated whether translocations of hihi (*Notiomystis cincta*), a threatened New Zealand passerine, achieved their implicit objective of restoring lost pollination function. Through a pollinator‐exclusion experiment, we quantified, with log response ratios (ln*R*), the effects of birds on fruit set and seed quality in hangehange (*Geniostoma ligustrifolium*), a native flowering shrub. We isolated the contributions of hihi by making comparisons across sites with and without hihi. Birds improved fruit set more at sites without hihi (ln*R* = 1.27) than sites with hihi (ln*R* = 0.50), suggesting other avian pollinators compensated for and even exceeded hihi contributions to fruit set. Although birds improved seed germination only at hihi sites (ln*R* = 0.22–0.41), plants at sites without hihi had germination rates similar to hihi sites because they produced 26% more filled seeds, regardless of pollination condition. Therefore, although our results showed hihi improved seed quality, they also highlighted the complexity of ecological functions. When an important species is lost, ecosystems may be able to achieve similar function through different means. Our results underscore the importance of stating and monitoring the ecological benefits of conservation translocations when functional restoration is a motivation to ensure these programs are achieving their objectives.

## Abstract 2

*Article Impact Statement*: Conservation translocations can help restore ecosystem function, but functional objectives must be stated and monitored to maximize success.

## Abstract (es)

Evaluación del Éxito de la Restauración Funcional Posterior a la Reintroducción de un Ave Polinizadora Desaparecida

**Resumen:**

La reubicación para la conservación es un método común para la recuperación de especies en el cual un objetivo cada vez más frecuente es la restauración de las funciones ecológicas que se perdieron para promover la recuperación del ecosistema. Sin embargo, pocos programas de reubicación para la conservación establecen o monitorean explícitamente a la función como un objetivo, lo que limita la posibilidad de comprobar suposiciones, aprender de esfuerzos anteriores y mejorar la gestión. Analizamos si las reubicaciones de hihi (*Notiomystis cincta*), un ave paseriforme amenazada de Nueva Zelanda, lograron el objetivo implícito de restaurar la desaparecida función de polinización. Mediante un experimento de exclusión del polinizador, cuantificamos con relaciones de respuesta logarítmica (ln*R*) los efectos de las aves sobre el conjunto de frutos y la calidad de la semilla del arbusto floral nativo *Geniostoma ligustrifolium*. Aislamos las contribuciones del hihi cuando comparamos entre sitios con y sin su presencia. Las aves favorecieron más al conjunto de frutos en sitios sin hihi (ln*R* = 1.27) que en los sitios con hihi (ln*R* = 0.50), lo que sugiere que otras aves polinizadoras compensaron y excedieron las contribuciones del hihi al conjunto de frutos. Aunque las aves aumentaron la germinación de semillas sólo en sitios con hihi (ln*R* = 0.22‐0.41), las plantas en los sitios sin hihi tuvieron tasas de germinación similares a los sitios con hihi porque produjeron 26% más de semillas completas sin importar la condición de la polinización. Por lo tanto, aunque nuestros resultados mostraron mejoras en la calidad de la semilla a causa del hihi, también resaltaron la complejidad de las funciones ecológicas. Cuando desaparece una especie importante, puede que los ecosistemas logren una función similar por medio de diferentes métodos. Nuestros resultados hacen hincapié en la importancia que tiene establecer y monitorear los beneficios ecológicos de las reubicaciones para la conservación cuando la restauración es motivo para asegurar que estos programas están logrando sus objetivos.

## INTRODUCTION

The decline of one species can have cascading effects on many others by disrupting important ecological functions (e.g., Brodie et al., 2014; Kelly et al., 2010; Soulé et al., 2003). This raises the stakes for conservation, but it also presents an opportunity to use interventions targeted at single species to achieve broader ecological objectives (Simberloff, 1998). Conservation translocations typically focus on species recovery but can also promote ecosystem recovery by restoring lost mutualisms, reintroducing keystone species, or introducing ecological replacements (e.g., Ewen et al., 2012; Seddon, 2010; Seddon et al., 2014). For example, seed dispersal has been restored through reintroduction of brown howler monkeys (*Alouatta guariba clamitans*) (Genes et al., 2018) and red‐rumped agoutis (*Dasyprocta leporina*) (Mittelman et al., 2020) in Brazil and ecological replacement of extinct giant tortoises by extant species in Mauritius (Griffiths et al., 2011) and the Galápagos (Hunter et al., 2013). Despite their restorative potential, translocation programs typically focus on how reintroduced species may harm the ecosystem (Armstrong & Seddon, 2008; Polak & Saltz, 2011). Only 6% of recently reviewed conservation translocations explicitly stated ecosystem restoration as an objective (Chauvenet et al., 2016; Seddon & Armstrong, 2019; Taylor et al., 2017), and those that did rarely evaluated whether this objective was achieved (Ewen et al., 2014).

Ecological function may be overlooked in conservation translocation planning because it is assumed to be a byproduct of species recovery. However, populations that meet common recovery benchmarks (e.g., minimum viable population size [Gilpin & Soulé, 1986; Shaffer, 1981]) are not always large enough to fulfill their ecological functions (e.g., Akçakaya et al., 2020; Conner, 1988; McConkey & Drake, 2006). Using these benchmarks to evaluate ecosystem recovery could, therefore, result in a “half‐empty forest” (Redford & Feinsinger, 2001), where species have technically recovered but key functions remain missing. Additionally, many threatened species declined so long ago that their ecological role is poorly understood (e.g., Anderson et al., 2016; Culliney et al., 2012; Gordon & Letnic, 2016), unachievable in the current environment, or occupied by another species that already repaired the functional deficiency (Akçakaya et al., 2020). These points are particularly relevant for rewilding initiatives, for which function is the primary motivation (Pettorelli et al., 2019; Seddon et al., 2014), but they apply to any conservation translocation that aims to restore function. If function is an objective, then stating this, designing actions to achieve it, and monitoring outcomes provides the best chance of success.

Animal‐mediated pollination is in crisis globally, threatening the stability of many ecosystems (Potts et al., 2010). Although conservation translocations have long been used to restore floral species threatened by pollinator declines (Abeli & Dixon, 2016), few have considered translocating the pollinators themselves (Cariveau et al., 2020) (but see van Winkel et al., 2010; LaBar et al., 2014; Sears et al., 2016). Conservation scientists may hesitate to translocate pollinators due to the relative costliness and complexity of manipulating animals (Dixon, 2009; Morton & Rafferty, 2017). Plant visitation does not guarantee high‐quality pollination (Bestea et al., 2019; Hervías‐Parejo & Traveset, 2018; King et al., 2013), so translocated species need to be selected carefully to ensure they visit target plants and pollinate them effectively. The ecological need for restoration must also be evaluated carefully because pollinator losses can be compensated by plant adaptations (Schleuning et al., 2016) or increased visitation by other pollinators (Hallett et al., 2017), including non‐native species (Pattemore & Wilcove, 2012).

Despite these challenges, conservation translocation will likely become necessary for restoring pollination as it becomes more difficult for extirpated pollinators to recolonize areas without assistance. This is evident in New Zealand, where rapid declines of native birds, due to disease, deforestation, and predation by introduced mammals, are associated with serious declines of native plants (Kelly et al., 2010). Historically, birds were thought to make only incidental contributions to pollination because the majority of native plants have an entomophilous flower syndrome (Clout & Hay, 1989; Godley, 1979). However, more recent studies show that several native birds are important (Anderson, 2003; Anderson et al., 2021; Kelly et al., 2010) or essential to pollination (Anderson et al., 2011). Translocations are frequently used to restore native bird populations, with many motivated at least in part by ecosystem restoration (Parker, 2013), but few actively test whether restoration is achieved.

The hihi (*Notiomystis cincta*) is an endemic passerine thought to have been an important pollinator in New Zealand before its widespread decline and near extinction in the late 1800s (Anderson, 2003; Anderson et al., 2011; Kelly et al., 2006). Hihi reintroductions are primarily aimed at species recovery but frequently cite the restoration of pollination function as an additional benefit (Ewen & Armstrong, 2007). However, no hihi reintroduction program has explicitly addressed this objective or evaluated whether it is achieved. Thus, uncertainty remains about the species’ role (How much do hihi contribute to pollination relative to other species?), ecological need (How significantly is pollination reduced at sites without hihi?), and reintroduction effectiveness (How successfully do hihi reintroductions restore pollination function?).

Evaluating success in achieving objectives requires monitoring appropriate metrics over a suitable time frame (Gregory et al., 2012). Although comparing ecological function immediately before and after a hihi translocation could help attribute functional changes to hihi, restoration may require more time to become evident (Choi, 2004; Pullin et al., 2013). Therefore, we employed an alternative strategy by comparing function across sites with and without hihi to evaluate their importance to the ecosystem (similar to the “elimination approach” [Akçakaya et al., 2020]). Focal hihi populations were established via reintroduction 12−22 years prior to our study and had reached relatively stable population densities (partially set by management effort) at least half that of New Zealand's only remnant hihi population (see Methods). We expected this time frame and density would be sufficient to yield measurable pollination benefits. Through a pollinator‐exclusion experiment, we quantified the contributions of bird and insect pollinators to three pollination outcomes: fruit set, filled seed set, and seed germination. Based on past assumptions about the benefits of hihi reintroductions, we expected plants at hihi sites to be less pollen limited (more maximally pollinated) and receive more pollination from birds than plants at sites without hihi.

## METHODS

### Study species

**FIGURE 1:**
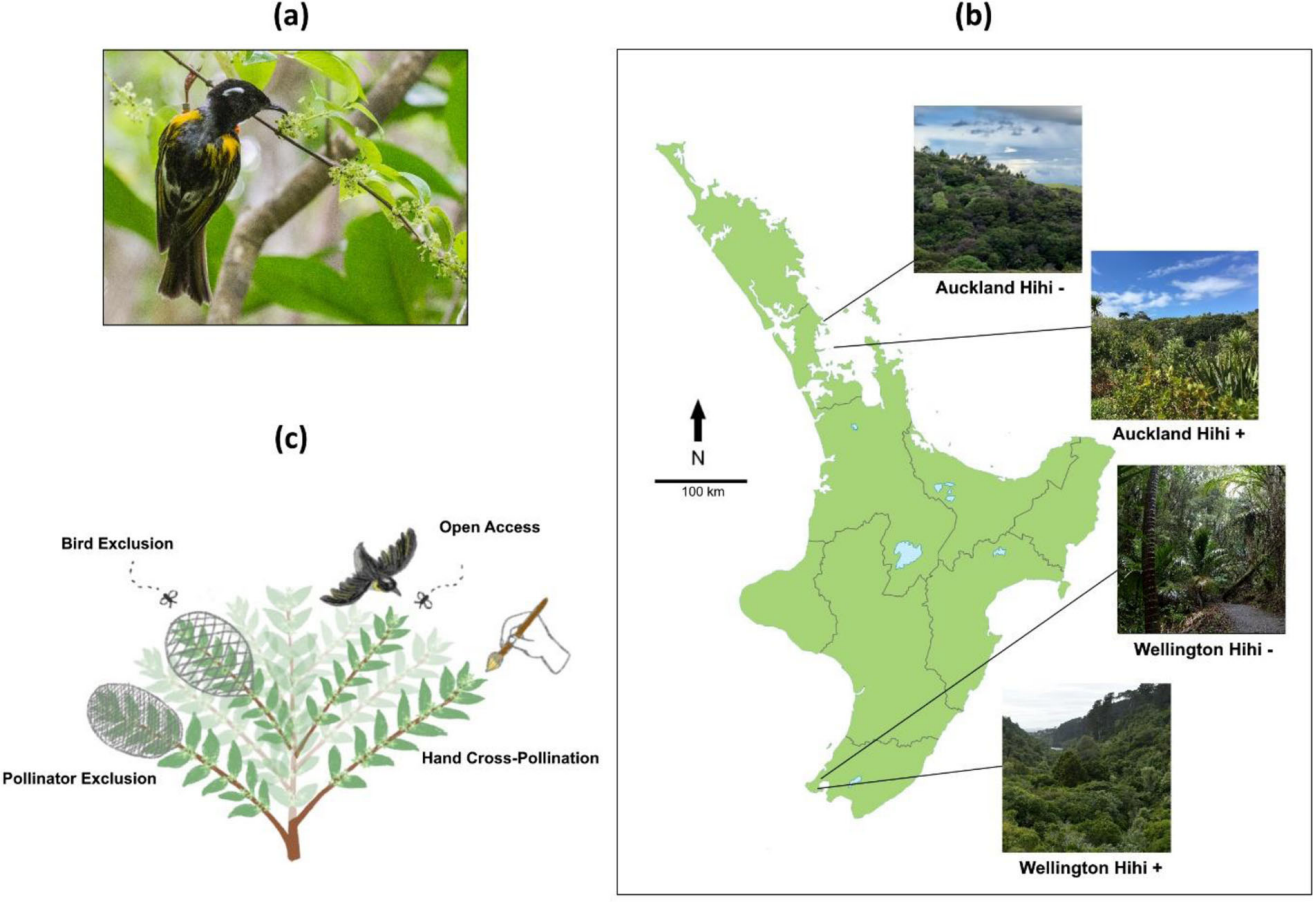
(a) A male hihi foraging on hangehange (photo by Martin Sanders), (b) locations of the four study sites on the North Island of New Zealand (Auckland hihi+ photo by C.A.; Auckland hihi− photo by Sharon Kast; Wellington hihi+ and hihi− photo by Christopher Stephens) (+, present; –, absent), (c) pollinator‐exclusion experimental design, showing a hangehange plant with branches exposed to the four pollination conditions: pollinator exclusion (all pollinators excluded by a fine mesh bag), bird exclusion (bird pollinators excluded by a 2 × 2 cm wire mesh cage), open access (branch uncovered), and hand cross‐pollination (branch uncovered and cross‐pollinated by hand during flower receptivity) (illustration by Rachel Moon)

The hihi is the sole member of the Notiomystidae family (Driskell et al., 2007; Figure 1a). Once widespread throughout New Zealand's North Island, they were reduced to a single remnant population on Te Hauturu‐o‐Toi (Little Barrier Island) (estimated population density 1.0 hihi/ha [Toy et al., 2018]) by 1890 and are classified as vulnerable (IUCN, 2017). Since the 1990s, translocations have established seven additional populations at sites where introduced mammalian predators have been excluded (Franks et al., 2019; Thorogood et al., 2013). All reintroduced populations depend on supplementary provisioning of sugar water (Chauvenet et al., 2012; Doerr et al., 2017; Thorogood et al., 2013) but prefer their natural diet of invertebrates, fruit, and nectar (Andrews et al., 2020; Rasch & Craig, 1988; Roper, 2012) when sufficiently available. Nectar use peaks at ∼56% of the hihi diet in spring (Rasch & Craig, 1988), coinciding with their breeding season and the flowering period for many native plants.

We focused on the mutualism between hihi and hangehange (*Geniostoma ligustrifolium*), a gynodioecious (Rattenbury, 1980) native understory shrub. Hangehange is abundant, making it amenable to cross‐site comparisons, but is nonetheless thought to be pollen limited on the New Zealand mainland (McNutt, 1998), suggesting a need for functional restoration. Its flowers form in inflorescences in spring and are small (<4 mm), scented, and pale green, so it was long assumed to be primarily insect pollinated. Known insect visitors include beetles (Coleoptera), flies (Diptera), butterflies and moths (Lepidoptera), and bees (Hymenoptera) (Anderson, 2003; Norton, 1984). It has since been shown to be visited frequently by birds, including hihi and two native honeyeaters, tūī (*Prosthemadera novaeseelandiae*) and korimako (*Anthornis melanura*). Among these, hihi are presumed the primary pollinators. Only hihi and korimako could meet their energetic demands foraging on hangehange (Castro & Robertson, 1997), and hihi visit hangehange more frequently (Roper, 2012) and probe more flowers per visit than korimako (Castro & Robertson, 1997). A fourth species, the tauhou (*Zosterops lateralis*), is a recently (c. 1856) self‐introduced native species that has been observed visiting hangehange more frequently than hihi at sites with both species (Burns, 2013). Past studies on hangehange focused on diurnal pollinators, so less is known about other potential nonavian vertebrate pollinators; however, the primary candidates (e.g., bats and geckos) are either rare or absent from all study sites.

#### Study sites

The study was conducted on New Zealand's North Island in two regions approximately corresponding to the northern (Auckland) and southern (Wellington) extremes of the hihi's historic range. Within each region, we selected one nature reserve containing hihi (hihi+) and a second that did not contain hihi (hihi−). Each site consisted primarily of regenerating broadleaf forest with small patches of remnant mature native bush. Paired sites were selected to be as ecologically similar as possible in habitat structure, presence of hihi predators (native, e.g., ruru [*Ninox novaeseelandiae*], and non‐native), and relative abundance of hangehange's avian pollinators and flower predators (Appendix S1). These species also represent some of the closest competitors of hihi. We assessed ecological similarity quantitatively during the study period. Briefly (detailed methods in Appendix S1), habitat assessments measured the density, diversity, and structure of vegetation at each site and confirmed that paired hihi+ and hihi− sites were indistinguishable, apart from a denser understory at the Auckland hihi+ site relative to the Auckland hihi− site. Observations of pollinator visitation confirmed that hihi were the primary avian visitors to hangehange at each hihi+ site (percentage of observed visits: Auckland hihi+ 67% and Wellington hihi+ 100%). We also observed visitation by korimako (Auckland hihi+ 33% and Auckland hihi− 100%;) but not by tauhou or tūī (Wellington hihi, no observed visits by any species). Anecdotal observations at the Auckland hihi+ site included destructive foraging by kākāriki (*Cyanoramphus novaezelandiae*), a known flower predator (Kelly et al., 2010).

The Auckland hihi+ site was Tiritiri Matangi Island (36°36'00.7“S 174°53'21.7”E; Figure 1b), a 220‐ha nature reserve 3.5 km offshore. After over a century of farming, the island was extensively replanted in the 1980s, and introduced mammalian predators were eradicated in 1993. Today, the island provides sanctuary to many native birds. A hihi population was established through translocation in 1995 and had ∼150 adults (0.68 hihi/ha) during our study. Located 25 km north, the Auckland hihi− site was Tāwharanui Regional Park (36°22'12.6“S 174°49'54.9”E) (Figure 1b). This 588‐ha peninsular reserve is considered a mainland island, a refuge for native wildlife that is surrounded by a fence to exclude introduced mammalian predators (Saunders & Norton, 2001). Tāwharanui's fence excludes all introduced mammals except mice.

Another mainland island, Zealandia Ecosanctuary (41°17'24.4“S 174°45'13.4”E) (Figure 1b), served as the Wellington hihi+ site. Located in Wellington city center, the 225‐ha fenced reserve contains a large reservoir and forest that has been regenerating since the early 1900s. Hihi were reintroduced in 2005, and there were ∼120 adults (0.53 hihi/ha) during our study. Approximately 15 km northeast, Belmont Regional Park (41°12'07.2“S 174°52'32.0”E) (Figure 1b) was the corresponding hihi− site. Although much larger than Zealandia (3500 ha), Belmont is dominated by pastureland with small patches of regenerating and remnant native bush. We conducted our study in the Korokoro Dam area, which is similar to Zealandia in its forest structure and history as a water catchment area. Belmont is unfenced, but site managers use bait stations and traps to control introduced mammalian predators. At the start of our study, tracking rates (percentage of tracking tunnels with evidence of mammalian predators) in the study area were ∼20% for rats and ∼22% for mice (Uys, 2017a, 2017b).

### Pollinator‐exclusion experiment

At each site, 30 mature hangehange plants were selected for a pollinator‐exclusion experiment. An additional 18 plants were included at the Auckland hihi+ site to account for the island's drier climate. We anticipated that some plants might fail to set fruit due to water stress (reducing our usable data set). Hangehange is gynodioecious, and hermaphrodite individuals set seed only rarely (Rattenbury, 1980), so we attempted to select only female plants based on the abundance of dry fruit capsules remaining from the previous reproductive cycle (plant selection occurred before the flowering season). To account for potential identification errors, we also quantified self‐pollination rates within each plant (see below).

Focal plants were distributed evenly across 10 habitat plots at each site (Auckland hihi+ 3−5 plants/plot; all other sites three plants/plot). At hihi+ sites, plots were on hihi breeding territories to ensure bird visitation was most likely by hihi, which chase intra‐ and interspecific intruders off their territories (Ewen et al., 2004; Low, 2005). Almost all hihi nest in nest boxes at both hihi+ sites, so we defined territories conservatively as a 20‐m radius around a hihi nest box. Plots were selected prior to the start of the hihi breeding season, targeting territories with a high chance of occupancy based on the past three breeding seasons. At hihi− sites, each 20‐m radius plot was chosen for its ecological similarity to hihi territories.

Following established methods (Kearns & Inouye, 1993), on each plant we assigned one branchlet containing at least 10 unopened buds to each of the following pollinator conditions (Figure 1c): pollinator exclusion, branchlet enclosed in a fine organza bag to exclude all pollinators; bird exclusion, branchlet enclosed in a 2 × 2 cm wire mesh cage to exclude birds but allow insects (mesh size selected following Anderson [2003]; continued insect visitation confirmed in Schmidt‐Adam et al. [2009]; no birds observed attempting to forage through mesh during observations); open access, branchlet left open to natural pollination; and hand cross‐pollination, branchlet left open to natural pollination and cross‐pollinated by hand during the receptive period (pollen gathered from 5 to 8 hangehange plants across the site and applied to flowers with a paintbrush, following Anderson et al. [2011]). This experimental design accounted for natural variation among plants by making the key comparisons between branches within the same plant. All sites were visited at the start of the flowering season in September 2017 to select branchlets, count buds, and apply coverings. Sites were revisited once flowers were open and receptive (approximately 3 weeks later) to complete hand pollination. Coverings were removed once all flowers passed receptivity (6−8 weeks after coverings first applied). Wellington sites were always visited ∼2−3 weeks after Auckland sites to account for latitudinal differences in phenology.

### Pollination outcomes

In late December 2017, fruits on each branchlet were counted and compared with the original number of buds to yield a measure of fruit set (proportion of buds that developed into fruits). For a subset of plants (11 per site), fruits were collected from the bird exclusion and open access conditions in mid‐February (Auckland) to early March (Wellington) and transported in paper bags to the Ōtari Native Botanic Garden in Wellington. Once capsules split open to release seeds, fruits were removed from the bags and rubbed on a paper towel to remove the sticky outer layer. Across all fruits from a branchlet, 80 seeds (where available) were selected randomly for germination testing (8 replicates of 10 seeds each). Where fewer seeds were available (13/56 branchlets), all seeds (mean [SE] = 33.6 [6.5]) were divided among replicates. Seeds were plated on 1% water agar in 90‐mm plastic petri dishes divided into five sections (one replicate per section). Dishes were incubated at 15 ° and 25 °C alternately in a respective 16‐ and 8‐h dark‐light cycle, inspected every 14 days for contamination and agar desiccation, and randomly repositioned in the incubator after each inspection.

At least 4 weeks after plating, seeds were inspected for germination (radical protrusion ≥1 mm [Appendix S2]). Ungerminated seeds were dissected and identified as filled if they contained a structured endosperm or unfilled if they were mushy or lacking a clear structure (Appendix S2). These data provided up to eight replicate measures per plant (Appendix S2) of three seed‐quality metrics: filled seed set (proportion of all seeds filled), germination probability of filled seeds (proportion of filled seeds that germinated), and germination probability of all seeds (proportion of all seeds that germinated).

### Data analyses

From our original data set of 138 plants, 31 were excluded due to total fruit set failure (open access and hand pollination conditions yielded no fruit). As predicted based on its drier climate, a greater proportion of plants (16 of 48) were excluded at the Auckland hihi+ site compared with the other sites (Auckland hihi−, 2 of 30; Wellington hihi+, 5 of 30; and Wellington hihi−, 8 of 30). The pollinator exclusion condition produced fruit in only 5 of the 107 remaining plants, so effects of self‐pollination were assumed negligible, and this condition was excluded from further analyses.

All analyses were conducted in R 4.0.0 (R Core Team, 2020). We used likelihood ratio tests to assess the significance of our main hypotheses in a stepwise fashion, in which we compared a model containing the interaction of interest (pollination condition∗hihi presence) against a simplified model without the higher‐order effect (pollination condition+hihi presence). If the interaction was not significant, we used *z* tests to test the significance of each factor in the additive model. For models testing fruit set as the pollination outcome, fruit count was the response variable with an offset of log(bud count), and negative binomial models were used to account for overdispersion (glmmTMB package) (Brooks et al., 2017). Models included region as a covariate and individual plant identity (ID), site, and territory as random effects to account for possible genetic differences (among plants and plant populations) and habitat and climatic effects (across sites and territories). For the seed‐quality metrics, response variables were proportions (accounting for the number of seeds in each replicate), and models were structured as generalized linear mixed models with a binomial family, logit link function, and bound optimization by quadratic approximation (BOBYQA) optimizer to reduce convergence errors. Models contained region as a covariate and random effects of site and plant ID (as above). Because multiple replicates from the same plant were plated on the same petri dish, a random effect of plate ID was included (nested within plant ID). An observation‐level random effect was included in filled seed set models to correct for overdispersion (Harrison, 2014).

All figures present predicted values from the minimal model. Predicted fruit counts were converted to proportions by dividing the original number of buds on each branchlet. Reported means for each condition were derived from the model predictions and are accompanied by their estimated standard errors. These means were also used to calculate a log response ratio (Knight et al., 2005) as a measure of pollen limitation at hihi+ and hihi− sites (ln*R*_hand_). The ln*R*_hand_ was calculated as:

(1) $$\ln\left(\frac{\text{fruit}\;\text{se}t_{\text{hand}\;\text{pollination}}}{\text{fruit}\;\text{se}t_{\text{open}\;\text{access}}}\right)$$

and indicates whether plants are pollen limited (ln*R*_hand_ > 0) or receive maximal pollination (ln*R*_hand_ ≤ 0). We used a similar approach to quantify the effects of birds on fruit set (ln*R*_bird_):

(2) $$\ln\left(\frac{\text{fruit}\;\text{se}t_{\text{open}\;\text{access}}\;}{\text{fruit}\;\text{se}t_{\text{bird}\;\text{exclusion}}\;}\right),$$

which indicates whether birds improve (ln*R*_bird_ > 0), reduce (ln*R*_bird_ < 0), or do not affect fruit set (ln*R*_bird_ = 0). The ln*R* values are presented only where the relevant conditions differed significantly in our models.

## RESULTS

**FIGURE 2:**
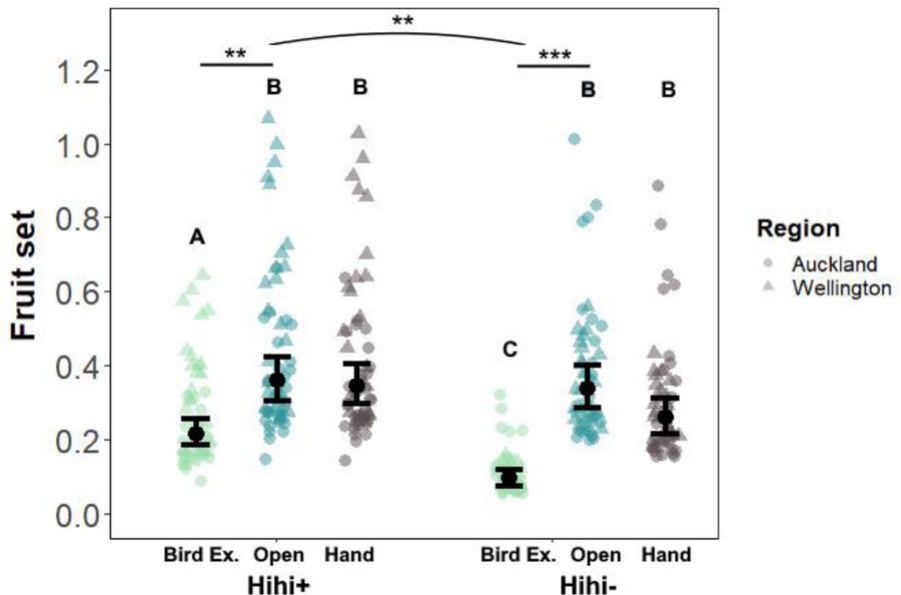
Differences in proportion of buds that set fruit relative to pollination condition (Bird Ex., bird exclusion; Open, open access; Hand, hand cross‐pollination) and hihi presence (+) or absence (−) (differing letters, significant differences across all means; stars, significant differences between conditions [lines] and interactions with hihi presence [arc]). Because region did not have a significant effect (Table 1), all points, means, and SEs (bars) are from a model without region as a covariate

The bird exclusion and open access conditions affected fruit set as expected; regardless of hihi presence, flowers set more fruit when exposed to birds (open access > bird exclusion) (Table 1 & Figure 2). If reintroducing hihi improves pollination, we would expect birds to improve fruit set more at hihi+ sites than hihi− sites. However, we found the reverse effect: a significant interaction between pollination condition and hihi presence (condition∗hihi: χ^2^ = 7.50, df = 2, *p* = 0.024) indicated that birds improved fruit set more at hihi− sites (ln*R*_bird_ = 1.27) than hihi+ sites (ln*R*_bird_ = 0.50) (Table 1 & Figure 2). Patterns of pollen limitation also deviated from expectation. We predicted that plants would be less pollen limited (more maximally pollinated) at hihi+ sites than hihi− sites, but hand pollination did not improve fruit set significantly at any site (Table 1 & Figure 2). Overall, fruit set patterns did not differ between regions (Table 1).

**TABLE 1:** Results of generalized linear mixed models in which pollination condition, hihi presence, and region predict fruit set, filled seed set, germination of filled seeds, and germination of all seeds for hangehange (*Geniostoma ligustrifolium*)

Predictor	Estimate^a^	SE	*z*	*p*
Fruit set				
intercept	−1.16	0.18	−6.31	<0.001
condition bird exclusion	−0.50	0.17	−3.01	0.003
condition hand pollination	−0.04	0.16	−0.24	0.814
hihi absent	−0.05	0.22	−0.23	0.815
region Wellington	0.29	0.19	1.55	0.122
condition bird exclusion, hihi absent	−0.78	0.29	−2.68	0.007
condition hand pollination, hihi absent	−0.23	0.24	−0.94	0.347
Filled seed set				
intercept	1.52	0.33	4.56	<0.001
condition bird exclusion	−0.49	0.09	−5.74	<0.001
hihi absent	1.25	0.39	3.19	0.001
region Wellington	−0.76	0.39	−1.96	0.051
Germination of filled seeds				
intercept	2.17	0.33	6.58	<0.001
condition bird exclusion	−0.74	0.15	−4.78	<0.001
hihi absent	−0.80	0.38	−2.11	0.035
region Wellington	−1.32	0.37	−3.58	<0.001
condition bird exclusion, hihi absent	0.69	0.22	3.13	0.002
Germination of all seeds				
intercept	0.90	0.33	2.74	0.006
condition bird exclusion	−0.62	0.10	−6.24	<0.001
hihi absent	−0.003	0.39	−0.01	0.994
region Wellington	−1.26	0.38	−3.30	<0.001
condition bird exclusion, hihi absent	0.48	0.16	2.98	0.003

^a^ Predictor estimates are from the minimal model identified through a likelihood ratio test (fruit set, germination of filled seeds, germination of all seeds: condition∗hihi+region; filled seed: condition+hihi+region). In all cases, results are presented for an intercept of open access pollination and Auckland hihi present.

**FIGURE 3:**
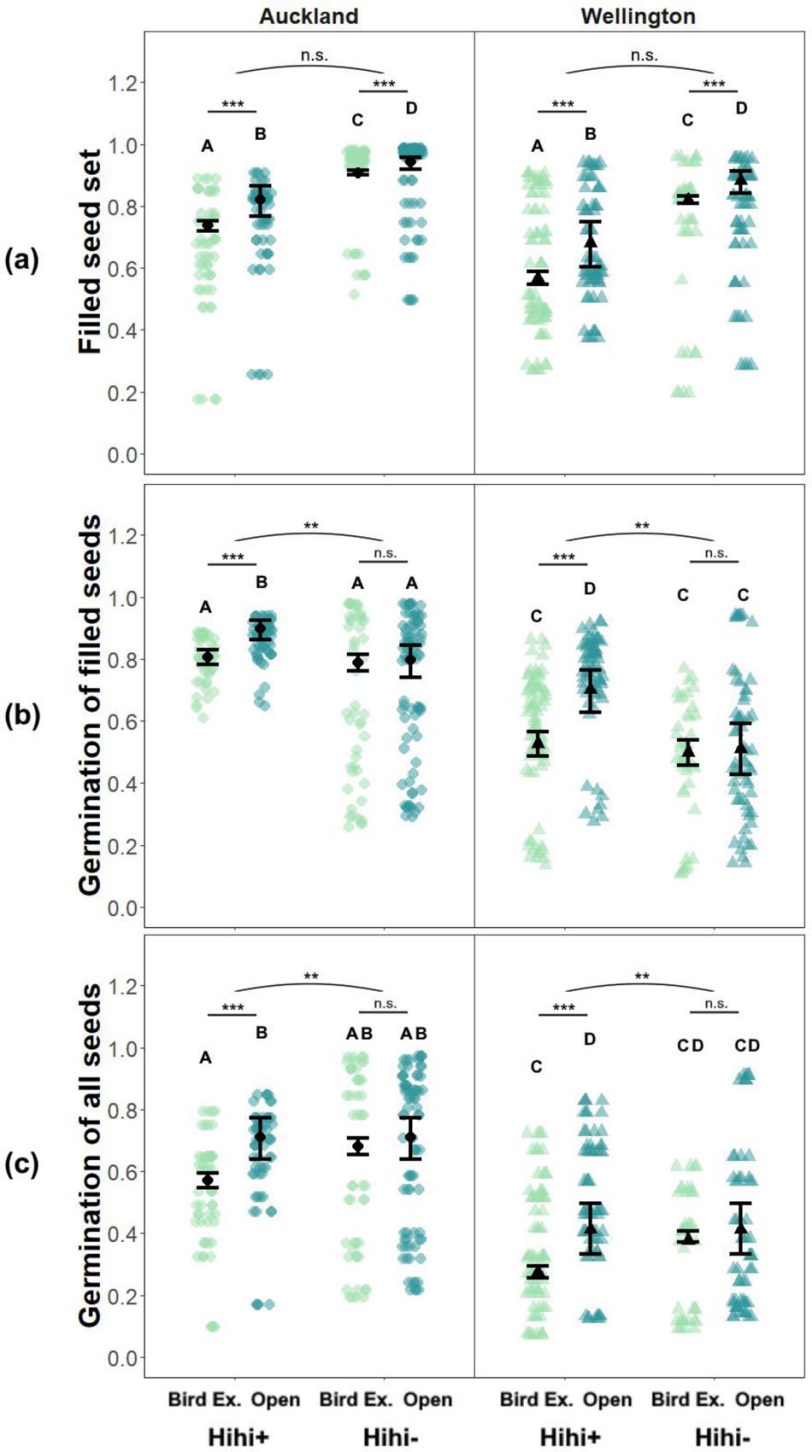
Differences in (a) filled seed set, (b) germination probability of filled seeds, and (c) germination probability of all seeds by pollination condition (Bird Ex., bird exclusion; Open, open access) and hihi presence (+) or absence (−) (differing letters, significant differences across all means; stars, significant differences between conditions [lines] and interactions with hihi presence [arcs]). Region either had a marginally nonsignificant (a) or significant (b−c: Auckland > Wellington) effect (Table 1), so all points, means, and SEs (bars) are from models with region as a covariate

Although only included in models to control for environmental differences, region had a significant effect on seed quality. Seed outcomes were significantly higher (germination rates) or trending higher (filled seed set) in Auckland than in Wellington, regardless of pollination condition. Results were mixed regarding the effect of hihi on these metrics. Flowers set a greater proportion of filled seeds when they received bird pollination (open access > bird exclusion), but birds improved filled seed set equally at hihi+ sites and hihi− sites (condition*hihi: χ^2^ = 0.03, df = 1, *p* = 0.87) (Table 1 & Figure 3a). This result contrasted with our prediction that hihi would improve seed quality more than other avian pollinators. Filled seed set was also significantly lower at hihi+ sites, regardless of pollination condition (Table 1 & Figure 3a). However, benefits of hihi visitation were evident in the germination rates of filled seeds (condition∗hihi: χ^2^ = 9.36, df = 1, *p* = 0.002). At hihi+ sites, birds improved germination of filled seeds significantly (ln*R*_bird_: Auckland 0.10 and Wellington 0.28) (Table 1 & Figure 3b). Meanwhile, at hihi− sites, germination of filled seeds was lower overall (Table 1), more variable (Figure 3b), and not significantly improved by birds (estimate = 0.04 [SE 0.16], *z* = 0.28, *p* = 0.78). The benefits of hihi remained when germination rates considered all (filled and unfilled) seeds (condition*hihi: χ^2^ = 8.80, df = 1, *p* = 0.003) (Table 1 & Figure 3c). Birds improved germination significantly at hihi+ sites (ln*R*_bird_: Auckland 0.22 and Wellington 0.41) but not hihi− sites (estimate = 0.14 [0.13], *z* = 1.12, *p* = 0.3). Nevertheless, because plants at hihi− sites produced a greater proportion of filled seeds at the outset, total germination rates were the same as at hihi+ sites (Table 1 & Figure 3c).

## DISCUSSION

In contrast to our predictions, fruit set was not pollen limited at sites with (hihi+) or without (hihi−) hihi, and birds improved fruit set more at hihi− sites. Plants at hihi− sites also produced a greater proportion of filled seeds, regardless of their exposure to birds. However, further analysis identified one benefit to hihi pollination: birds improved the germination of filled seeds only at hihi+ sites. Despite this benefit, the lower prevalence of filled seeds meant that overall germination rates were similar to hihi− sites.

Our results demonstrate that achieving valuable species recovery or biodiversity objectives with conservation translocation does not always translate directly into functional restoration. We found that objectives for hihi recovery and site biodiversity were achieved at each hihi+ site (i.e., a hihi population was reestablished), but this did not improve pollination function for our focal plant species, hangehange. This could reflect a flaw in two common assumptions of hihi translocations. First, hihi may be less effective pollinators than assumed (Ewen & Armstrong, 2007), either because their historic role was overestimated or their efficacy is reduced under current conditions. Similar studies on Australian seed dispersers demonstrate the challenges of inferring species’ historic roles from their performance in degraded landscapes with altered species assemblages (Gordon & Letnic, 2016; Mills & Letnic, 2018). Second, hihi may be effective pollinators, but restoring function may not be as simple as reintroducing them to the ecosystem. Elsewhere in New Zealand, controlling introduced predators has increased native pollinator populations without demonstrably improving function (Kelly et al., 2005; Anderson et al., 2021 [but see Iles & Kelly, 2014; Bombaci et al., 2021]); some evidence suggests that pollinator numbers were too low to achieve full functionality (Kelly et al., 2005). It is possible our assumption was incorrect that hihi populations had reached sufficient densities to improve pollination; if so, further management may be needed to promote population growth, and our results could provide a baseline against which to compare future functional improvements.

Equating biodiversity with ecological function risks oversimplifying ecosystem complexity. Fully biodiverse communities often include species with mutualistic and antagonistic roles (Bronstein et al., 2003), whose effects on function may cancel out. This could provide another explanation for the unexpected similarity between hihi+ and hihi− sites. Sites that have undergone sufficient restoration to support hihi (Ewen & Armstrong, 2007) may also be more hospitable to flower predators, such as kākāriki (Ortiz‐Catedral et al., 2009; Ortiz‐Catedral & Brunton, 2010), whose destructive foraging (as observed at the Auckland hihi+ site) may mask some of the benefits of hihi. Similarly, other restored islands in New Zealand contain high densities of native seed‐predating caterpillars, which may offset the effects of pollinators (Molloy, 2004). Furthermore, just as ecosystems change with restoration, degraded ecosystems are also dynamic and may be able to recover pollination function through increased visitation by other native (Hallett et al., 2017) or introduced species (Pattemore & Wilcove, 2012; Stavert et al., 2018; O'Rourke et al., 2020). Although not captured by our observations, compensatory visitation could explain why birds were so important to fruit set at hihi− sites and why fruit set was not pollen limited at any site (in contrast to McNutt, 1998). Situations like these may call into question how one views restored ecosystems if restoring biodiversity promotes both mutualistic and antagonistic interactions and degraded sites can achieve functionality through other means.

Although ecosystems may be able to compensate for the loss of a mutualist, measuring function across multiple levels can expose the limitations of these compensatory mechanisms. Despite the unexpected patterns in fruit set, we identified one potential benefit of hihi pollination: birds improved seed germination, but only at hihi+ sites. Thus, even if another avian pollinator can compensate for (and even exceed) the effects of hihi on fruit set, their benefits disappear at later stages of reproduction. This pattern can arise when plants shift their reproductive investment in the face of low‐quality pollination. They may still set fruit and seed (Craig & Stewart, 1988; Winsor et al., 1987; Vaughton & Carthew, 1993), but resulting seeds may be of lower quality (Stephenson, 1981) or exhibit reduced seedling growth and survival (Schmidt‐Adam et al., 2000; Robertson et al., 2011). The functional similarity observed across sites may, therefore, reflect a shift in investment by hangehange from quality to quantity in the absence of hihi, rather than the effective replacement of hihi. Given the contrasting effects of hihi on fruit set and seed quality, it could be that hihi reintroductions provide little net benefit. However, species can compensate for their deficiency at one stage of an ecological process by benefiting a more consequential stage. For example, in Bolivian forests, the purplish jay (*Trochocercus cyanomelas*) does not disperse seeds as far as the chestnut‐eared araçari (*Pteroglossus castanotis*), but it brings a greater net benefit to plant populations by improving seedling emergence and depositing seeds in higher quality habitats (Loayza & Knight, 2010). Further work may be needed to trace the effects of hihi through to later stages of plant recruitment.

Overall, our results raise important questions about how to balance and assess multiple objectives in conservation translocations. Many, if not all, translocation programs are driven by multiple objectives, but constraints on monitoring often mean only one objective is evaluated and used as a proxy for the others. Our results highlight both the promises and pitfalls of using species establishment as a proxy for functional restoration. Reintroducing a lost avian pollinator brought some measurable benefits to ecological function, but the net benefits to the ecosystem were less clear. Most ecological systems have a degree of resilience. When the providers of a function are lost, the receivers may be able to compensate, such that impaired ecosystems may be less impaired than assumed. The difficulty of predicting, and then monitoring, one species’ contributions to the ecosystem may explain why translocation programs often rely on proxies to measure function. Yet, obtaining accurate measures of function is becoming more essential as ecological restoration becomes a higher priority in many translocation programs, including reintroductions, reinforcements, and ecological replacements (often as part of rewilding) (e.g., Seddon, 2010; Ewen et al., 2012; Seddon et al., 2014). We, therefore, encourage translocation programs to monitor this objective directly when possible and to select metrics carefully so success can be evaluated and improved through adaptive management (Canessa et al., 2016).

## Supporting information



Supplementary material

Figure S2‐1. Germination and filled seed set tests were performed on seeds collected from a subset of plants in the pollinator exclusion experiment.
Table S2‐1. Number of replicates (with the number of unique plants from which the replicates were derived in parentheses) tested by site and pollination condition for each of the three seed quality metrics.
Appendix S1. Site Assessments
