# Supplementary material for: Evaluating the success of functional restoration after reintroduction of a lost avian pollinator
Source: Conserv Biol. 2022 Apr 7;36(4):e13892. doi: 10.1111/cobi.13892 (PMC9545379; doi:10.1111/cobi.13892)
Supplement: Supplementary file 1 — Supplementary material [file COBI-36-0-s001.pdf]

SUPPORTING INFORMATION

Appendix S1: Site Assessments

Methods

Site Assessments

The two hihi+ sites (Auckland: Tiritiri Matangi Island [36°36'00.7"S 174°53'21.7"E]; Wellington: Zealandia Ecosanctuary [41°17'24.4"S 174°45'13.4"E]) were selected for this study based on their high hihi densities and ease of monitoring birds and plants. Site managers provided advice on which nearby nature reserves without hihi would be most similar in forest structure and pollinator assemblages (Table S1-1), leading to the selection of the two hihi− sites (Auckland: Tāwharanui Regional Park [36°22'12.6"S 174°49'54.9"E]; Wellington: Belmont Regional Park [41°12'07.2"S 174°52'32.0"E]).

To confirm the ecological similarity of sites within regions (and compare across regions), we conducted habitat assessments at each site on the plots containing plants for our pollinator exclusion experiment (see main text, Methods). Hangehange and foraging hihi are generally found in the understory (Gravatt, 1971; Roper, 2012), so habitat assessments focused on understory vegetation. On each plot, we designated five 20-m transects radiating evenly from a central point. At five sampling points along each transect, we counted and taxonomically identified every mature plant intersecting an imaginary cylinder of 1-m radius extending from the ground to a height of 2 m. Summing these counts across all 25 sampling points and dividing by the total sampling area

(25π m^2) provided an estimate of understory density and hangehange density. To quantify species richness and evenness, we calculated a Shannon diversity index (Shannon, 1948) as:

$$-\sum_{i=1}^{R} p_i ln p_i,$$

where R is the total number of unique species present and p_i is the proportion of plants belonging to species i. Lastly, we completed a vertical habitat assessment (adapted from Scott, 1965; Makan et al., 2014) at each sampling point by extending an imaginary cylinder of 10-cm radius from the ground to the canopy, identifying all species with intersecting foliage, and estimating the canopy height. Combined with the data from our understory assessments, this provided a count of the total number of unique species and an average canopy height for the habitat plot.

Pollinator visitation rates were assessed at each site during five hour-long sessions conducted during the period of hangehange flower receptivity. Observations were conducted over three days at the Auckland hihi+ site and one day at all other sites, with sessions spread evenly throughout the morning and afternoon. During each session, an observer sat quietly under cover of vegetation on one of the habitat plots with five mature hangehange plants in view and at least 5 m away. Each bird that visited one of the plants and foraged (probed or consumed flowers) was counted and identified to species level. At the end of the session, the number of flowers on each plant was estimated by multiplying the number of open flowers (estimated to the nearest 10) on one branchlet by the number of branchlets (estimated to the nearest 5) on the plant. These counts were used to calculate a visitation rate for each bird species, which was standardized to visits per hour per 10,000 flowers. Hangehange visitation outside of these observation sessions was noted anecdotally to capture visitation by additional species.

Data Analysis

We first conducted a Principal Component Analysis (PCA) to quantify the ecological similarity of the four study sites. Variables measured on habitat plots were pooled across sites and standardized prior to analysis to account for different scales. The minimum number of components needed to explain at least 70% of the variance were assessed visually using biplots, with convex hulls drawn around all datapoints (habitat plots) belonging to each site. We then used the vegan package (Oksanen et al., 2019) to test site differences quantitatively with a permutational multivariate analysis of variance using Euclidean distance matrices (Anderson, 2001). As this analysis indicated significant differences among sites (see Appendix S1, Results), post-hoc pairwise comparisons between sites were performed using a pairwise permutation MANOVA (PERMANOVA) with 1,000 permutations (RVAideMemoire package: Hervé, 2020). Our study design assumed paired hihi+ and hihi− sites to be ecologically similar; to avoid increasing the likelihood of Type II errors, we did not correct the resulting *P*-values, so results may overestimate differences among sites.

While PCA is useful for visualizing and quantifying the degree of similarity between groups, it can make it difficult to interpret which variables are responsible for group differences (Jolliffe & Cadima, 2016). Therefore, we followed our PCA with a conditional inference tree analysis. The ctree function (party package: Hothorn et al., 2020) performs a binary recursive partitioning procedure. Here, we used it to test the dependence of site on each of the habitat variables, performed a binary split of the data based on the variable with the strongest significant association with site, and continued until site was no longer associated with any of the remaining variables

(Hothorn et al., 2006). In this way, we could determine which sites were statistically distinguishable based on habitat and which variables explained site differences.

Results

The Principal Component Analysis identified strong associations between habitat variables measured across all sites. PC1 and PC2 explained a cumulative 70% of the variance among habitat plots (Table S1-2). All habitat variables loaded positively on PC1, with total species and hangehange density having the strongest loadings. Three of the five variables loaded positively on PC2, but understory density and total species had negative loadings. A biplot of PC1 and PC2 (Fig. S1-1a) identified a strong positive correlation between canopy height and Shannon diversity, and a negative relationship between these variables and understory density.

The habitat differed significantly across sites (ADONIS: $R^2 = 0.45$, $p < 0.001$). Although paired hihi+ and hihi− sites were selected to be as ecologically similar as possible, only the two Wellington sites could not be distinguished (PERMANOVA: $p = 0.060$; Auckland sites: $p = 0.001$). A visualization of the PCA (Fig. S1-1a) identified understory density as the primary difference between the Auckland sites, which was confirmed by the conditional inference tree analysis: the Auckland hihi+ site had a consistently denser understory (>0.993 plants/m^2; $p < 0.001$) than any other site (Fig. S1-1b). Meanwhile, the two Wellington sites were distinguished from the Auckland hihi− site by their lower canopy (≤6.92 m; $p = 0.002$; Fig. S1-1b).

Plant observations confirmed that hangehange visitation rates (standardized to visits per hour per 10,000 flowers) were higher at each hihi+ site compared with its corresponding hihi− site and that hihi were the primary visitors where they were present. At the Auckland hihi+ site, hihi visited hangehange twice as often (0.66 visits/h) as korimako (0.33 visits/h), and kākāriki were anecdotally observed foraging destructively on hangehange flowers (but their visitation was not captured during systematic plant observations). Visitation at the Auckland hihi− site was less frequent and was restricted to korimako (0.08 visits/h). In Wellington, only hihi were observed visiting hangehange at the hihi+ site (0.11 visits/h), while no visits by any species were observed at the hihi− site.

Tables and Figures

Table S1-1. Estimated relative abundances of hangehange’s avian pollinators and a known flower predator (kākāriki) at the four study sites.*

	Auckland		Wellington	
	Hihi+	Hihi−	Hihi+	Hihi−
Hihi	0.18	0.00	0.18	0.00
Korimako	0.53	0.54	0.02	0.02
Tūī	0.13	0.35	0.62	0.70
Tauhou	0.01	0.10	0.10	0.28
Kākāriki	0.15	0.01	0.08	0.00

*Values reflect the number of times each species was seen or heard in transect surveys (Auckland hihi+: Feb.–Mar. 2018, Stewart & Milton, 2018; Wellington hihi−: Sept. 2013, Greater Wellington Regional Council, unpublished data) or five-minute bird counts (Auckland hihi−: Jan. 2018, Maitland & Lovegrove [Auckland Council], unpublished data; Wellington hihi+: Sept. 2013, Zealandia Ecosanctuary, unpublished data) as a proportion of the total times any of the five focal species were seen or heard.

Table S1-2. Results of the Principal Component Analysis (PCA) of habitat variables measured on habitat plots across the four study sites (PC4 and PC5 not presented).

	PC1	PC2	PC3
Total species	0.58	−0.25	0.09
Understory density	0.39	−0.70	−0.06
Shannon diversity	0.39	0.46	0.70
Hangehange density	0.49	0.15	−0.09
Canopy height	0.36	0.47	−0.70
Eigenvalue	2.30	1.20	0.74
Proportion of variance explained	0.46	0.24	0.15

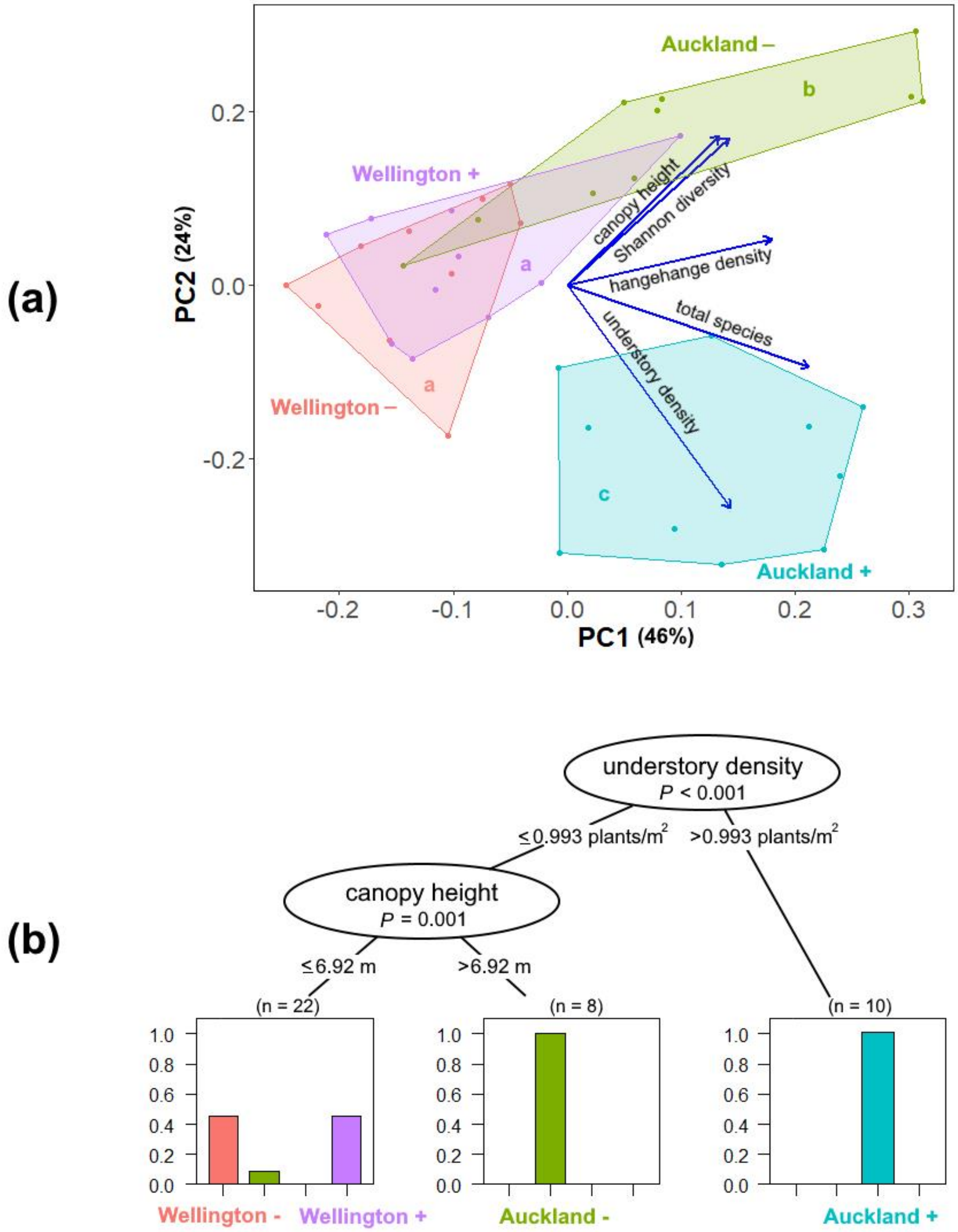

Figure S1-1. Habitat comparisons across the four study sites. Sites are labeled by region and hihi presence (+) or absence (−). (a) Biplot of the first two components from the Principal Component Analysis (PCA), with axes labeled with the percentage of variance explained by the component. Arrows represent the loadings for the habitat variables, which have been scaled prior to analysis. Points represent habitat plots grouped by site (shaded polygons). Letters within polygons indicate

significant differences among sites (PERMANOVA). (b) Results of a conditional inference tree analysis confirming ecological similarity between the two Wellington sites and a significant difference based on understory density between the two Auckland sites. *P*-values within each oval indicate the significance of each binary split of the data. Histograms indicate the proportion of data points (y-axis) at the terminal node belonging to each site (x-axis).

Literature Cited

Anderson, S. H. (2003) The relative importance of birds and insects as pollinators of the New Zealand flora. *New Zealand Journal of Ecology* 27(2), 83–94.

Gravatt, D. J. (1971) Aspects of habitat use by New Zealand honeyeaters, with reference to other forest species. *Emu* 71(2), 65.

Hervé, M. (2020) RVAideMemoire: Testing and plotting procedures for biostatistics. R package version 0.9-77. Available at: https://cran.r-project.org/

Hothorn, T., Hornik, K., Strobl, C., & Zeileis, A. (2020) party: A laboratory for recursive partitioning, R package version 1.3-5. Available at: https://cran.r-project.org/

Hothorn, T., Hornik, K., & Zeileis, A. (2006) Unbiased recursive partitioning: A conditional inference framework. *Journal of Computational and Graphical Statistics* 15(3), 651–674.

Jolliffe, I. T., & Cadima, J. (2016) Principal component analysis: a review and recent developments. *Philosophical Transactions of the Royal Society A: Mathematical, Physical and Engineering Sciences* 374(2065), 20150202.

Makan, T., Castro, I., Robertson, A. W., Joy, M. K., & Low, M. (2014) Habitat complexity and management intensity positively influence fledging success in the endangered hihi (Notiomystis cincta). *New Zealand Ecological Society* 38(1), 53–63.

Oksanen, J., Guillaume Blanchet, F., Friendly, M., Kindt, R., Legendre, P., McGlinn, D., Minchin, P. R., O'Hara, R. B., Simpson, G. L., Solymos, P., Stevens, H. H., Szoecs, E., & Wagner, H. (2019) vegan: Community Ecology Package. R package version 2.5-6. Available at: https://cran.r-project.org/

Roper, M. (2012) Resource partitioning between two competitive species, the hihi (Notiomystis

cincta) and bellbird (Anthornis melanura), during the non-breeding season on Tiritiri Matangi Island (thesis). Albany: Massey University.

Scott, D. (1965) A height frequency method for sampling tussock and shrub vegetation. *New Zealand Journal of Botany* 3(4), 253–260.

Shannon, C. E. (1948) A mathematical theory of communication. *The Bell System Technical Journal* 27, 623–656.

Stewart, J., & Milton, K. (2018) Tiritiri Matangi Island transect bird survey: 2018 report. *Supporters of Tiritiri Matangi Island*. Available at: http://www.tiritirimatangi.org.nz/
