# Supplementary material for: Evaluating the success of functional restoration after reintroduction of a lost avian pollinator
Source: Conserv Biol. 2022 Apr 7;36(4):e13892. doi: 10.1111/cobi.13892 (PMC9545379; doi:10.1111/cobi.13892)
Supplement: Supplementary file 2 — Figure S2‐1. Germination and filled seed set tests were performed on seeds collected from a subset of plants in the pollinator exclusion experiment. Table S2‐1. Number of replicates (with the number of unique plants from which the replicates were derived in parentheses) tested by site and pollination condition for each of the three seed quality metrics. Appendix S1. Site Assessments [file COBI-36-0-s002.docx]

SUPPORTING INFORMATION

Appendix S2: Seed Analysis Methodology


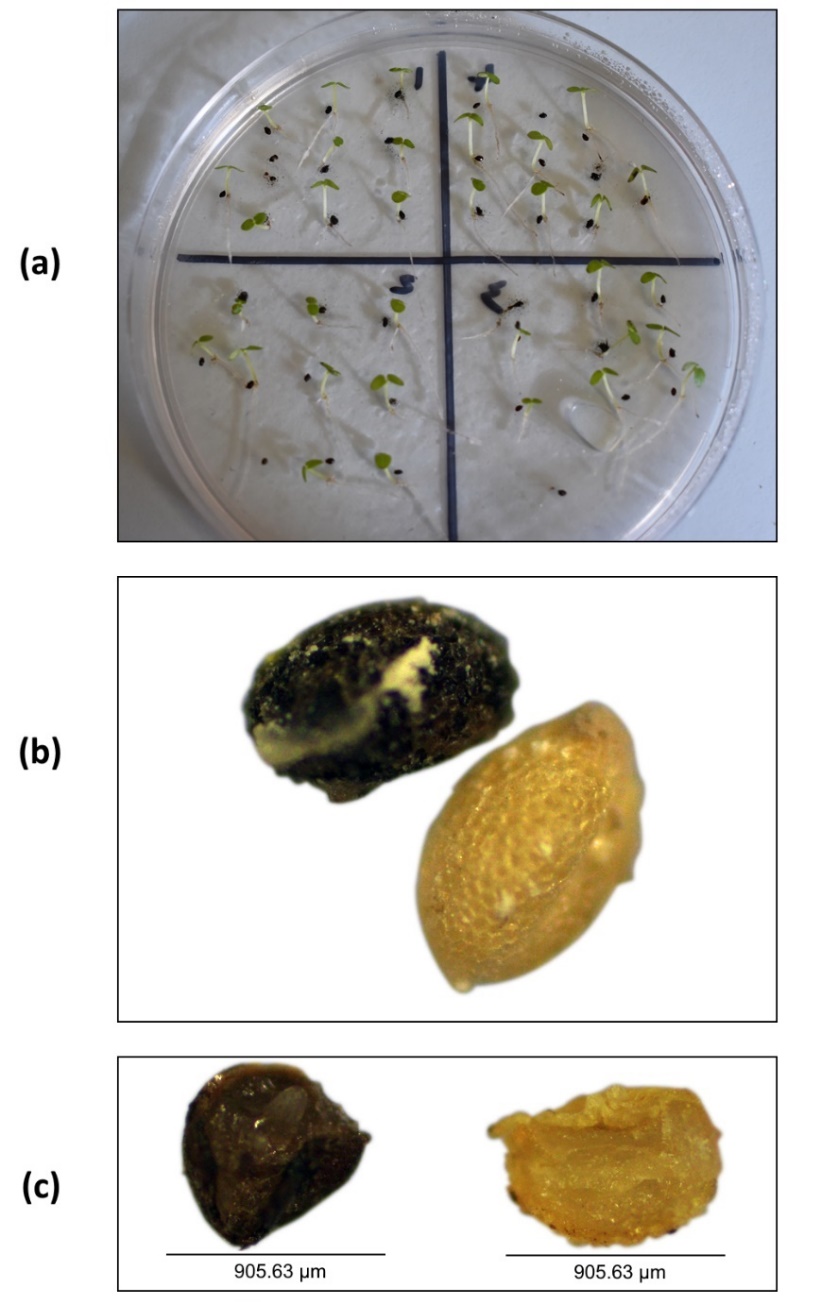


Figure S2-1. Germination and filled seed set tests were performed on seeds collected from a subset of plants in the pollinator exclusion experiment. (a) Seeds plated in a petri dish containing agar for the germination test, close to the date that germination was assessed (at least four weeks after plating). (b) Undissected filled seed (left) and unfilled seed (right). (c) Cross-section of a dissected filled seed (left) and unfilled seed (right). Photo credits: Karin van der Walt

Table S2-1. Number of replicates (with the number of unique plants from which the replicates were derived in parentheses) tested by site and pollination condition for each of the three seed quality metrics.

|  |  | Auckland | | Wellington | |
| --- | --- | --- | --- | --- | --- |
| Seed quality metric(s) | Pollination condition | Hihi+ | Hihi− | Hihi+ | Hihi− |
| Filled seed set, germination of all seeds | Bird exclusion | 53 (8) | 49 (7) | 78 (10) | 41 (6) |
|  | Open access | 58 (9) | 80 (11) | 68 (11) | 60 (8) |
|  |  |  |  |  |  |
| Germination of filled seeds* | Bird exclusion | 49 (7) | 49 (7) | 75 (10) | 41 (6) |
|  | Open access | 58 (9) | 80 (11) | 68 (9) | 60 (8) |

* Some values are lower for germination of filled seeds because this metric could not be assessed for replicates containing no filled seeds.
